# Effect of Polymer Matrix Type and Particle-Size Distribution on the Mechanical, Microstructural, and Thermal Behavior of Waste Tire Rubber Composites

**DOI:** 10.3390/polym18202599

**Published:** 2026-10-21

**Authors:** Mehrzad Mohabbi, Canan Kaya Sahin

**Affiliations:** Department of Civil Engineering, Faculty of Engineering, Bingöl University, 12000 Bingöl, Türkiye; canankaya231986@gmail.com

**Keywords:** waste tire rubber, polymer composites, epoxy, polyurethane, novolac–hexamine, unsaturated polyester, particle-size distribution, structure-property relationships

## Abstract

Recycling ground tire rubber (GTR) into polymer-matrix composites requires accounting for both matrix formulation and rubber particle-size distribution. Here, polyurethane, epoxy, novolac–hexamine, and unsaturated polyester systems were compared using four gradations of the same rubber source (16 formulations). A common main-resin-to-rubber mass ratio of 20% was used; differences in required auxiliary components and curing conditions were explicitly considered. The 28-day reference compression results ranged from 1.22 to 1.35 MPa for the polyester formulations to 8.21 MPa for epoxy–K1; epoxy–K1 also reached 2.21 MPa in flexure, whereas polyurethane–K4 reached 6.29 MPa in compression and 1.94 MPa in flexure. Thirteen available compression curves were reprocessed using the 70 × 50 mm loaded face and 70 mm loading length; derived deformation and work-density parameters are apparent, machine-displacement-based measures. Binder-specific UPV trends were exploratory (four gradations per binder). Selected SEM/EDS, ATR-FTIR, and representative TGA/DTG data supplied complementary evidence without establishing interfacial chemical bonding, cure inhibition, or fire performance. The results indicate formulation-dependent strength–deformation trade-offs within the tested materials rather than a universal optimum matrix or gradation.

## 1. Introduction

End-of-life tires are a persistent waste stream because their vulcanized rubber phase is crosslinked and not readily remelted. Mechanical processing produces granules that can be reused as a secondary feedstock in construction-related products [1,2,3]. However, rubber is a compliant, compositionally complex phase and cannot be assumed to increase composite strength. In cement-based materials, replacing mineral aggregate with rubber often reduces compressive strength while altering density and deformation behavior [4,5].

Polymer-bonded rubber composites differ fundamentally from rubber-modified concrete. The vulcanized tire rubber remains a pre-existing crosslinked particulate phase, while the separately introduced polymer matrix provides interparticle continuity. Matrix viscosity, polarity, reactive components, stiffness, and curing conditions may therefore affect performance. Work on thermoset rubber composites and flexible polyurethane tiles demonstrates that formulation and interface design are important alongside rubber loading [6,7,8].

Particle-size distribution affects the available interfacial area and the arrangement of granules. Smaller particles increase the area requiring polymer coverage; broader gradations may facilitate packing, although the irregular and compliant nature of rubber prevents straightforward transfer of mineral-aggregate assumptions. The direction of these effects depends on the actual polymer system, as reported in studies of polymer–rubber composites [2,7,9]. Fine, medium, and coarse rubber fractions must therefore be identified quantitatively rather than treated as universal categories.

Previous studies provide information about particular rubber–matrix combinations, including the deformation of polyurethane-based products, epoxy–rubber interfaces, and polyester–rubber composites [7,10,11,12,13,14,15,16,17]. Nevertheless, differences in rubber source, formulation, additives, processing, and analytical coverage limit direct comparisons among four distinct thermoset-related matrices. Furthermore, correlations between UPV and strength, local microscopy, and thermogravimetric behavior answer different questions and should not be treated as mutually validating proof of a particular reaction mechanism.

This study compares four polymer matrix formulations using one rubber source and four gradations: K1, 0.5–1.2 mm (G1); K2, nominal 5 mm (G2); K3, equal masses of G1 and G2; and K4, equal masses of G1–G4, where G3 is 5–10 mm and G4 is 10–20 mm. The objective is to identify matrix- and gradation-dependent tendencies in compression, flexure, UPV, and apparent machine-displacement-derived deformation, and to contextualize them with selected SEM/EDS, FTIR, and TGA/DTG observations. Our working hypothesis is that apparent strength and deformation trends depend on the combined effects of formulation and gradation; no universal optimal gradation or full-factorial chemical characterization is claimed.

Additional studies of crumb-rubber epoxy overlays, rubber-surface treatment, polyurethane-based repair mortars, and pretreated rubber/polyurethane foams illustrate how morphology, formulation, and processing can jointly affect performance [18,19,20,21]. These systems differ in matrix composition and experimental design; their results are used as context, not direct mechanistic proof for the present formulations.

Fire-retardant GTR–polyurethane foam studies [22,23] emphasize that thermogravimetry is distinct from standardized reaction-to-fire testing. Tire rubber has also been investigated in geopolymer mortars [24] and hybrid cementitious composites [25]. Rubber–phenolic resin interactions [26] and the review of surface-modification strategies for tire rubber [27] provide relevant contextual mechanisms, but neither independently demonstrates a particular interface reaction in the present samples. These studies concern different matrices and experimental designs and do not establish chemical curing mechanisms in the present materials. Here, reported effects are distinguished from possible explanations and applications requiring further tests.

## 2. Materials and Methods

### 2.1. Experimental Matrix, Rubber Fractions, and Formulation Basis

**Figure 1:**
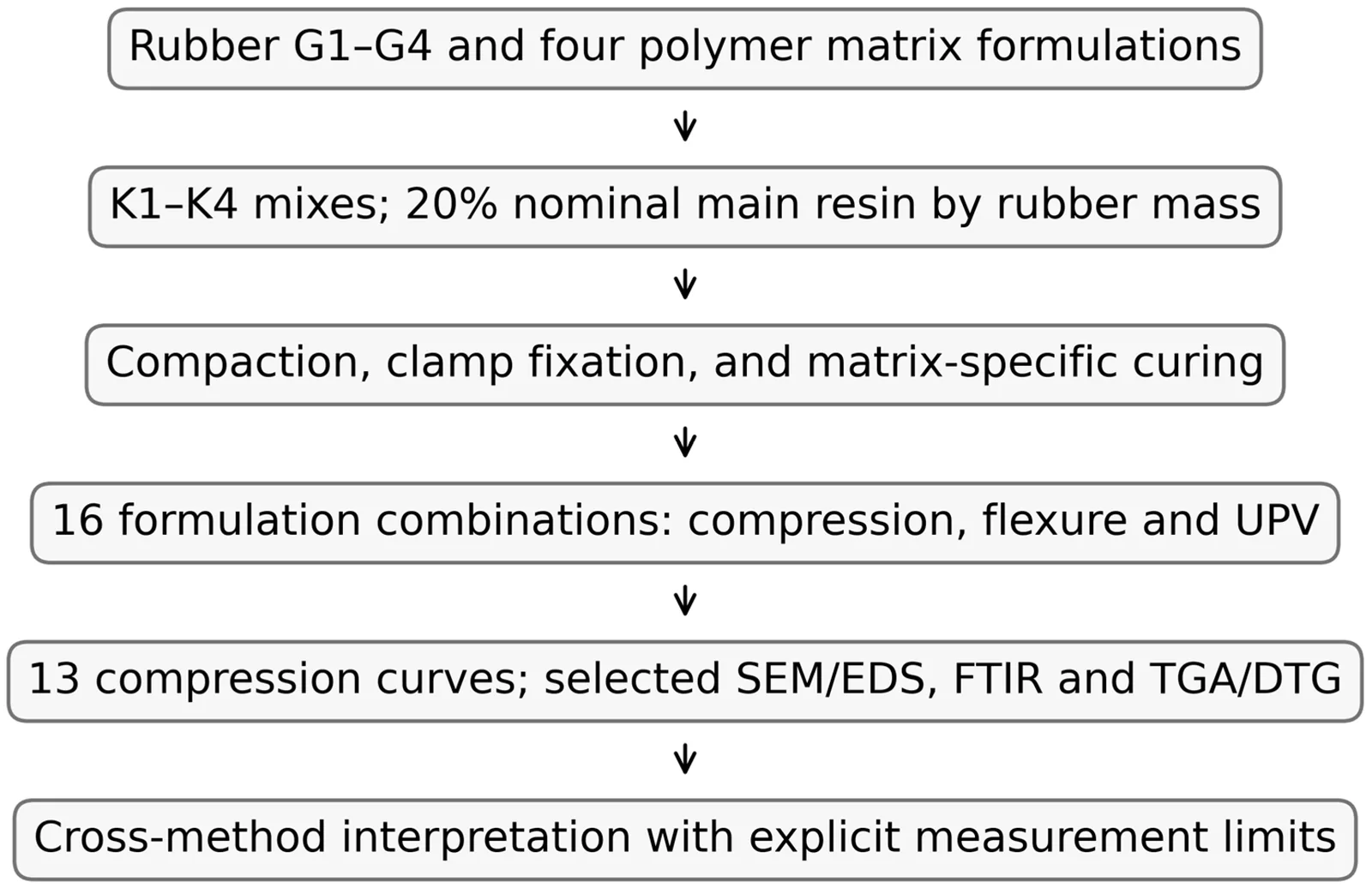
Simplified sequence of formulation, molding, mechanical testing, and selected characterization (method coverage is not identical for all 16 formulations).

**Figure 2:**
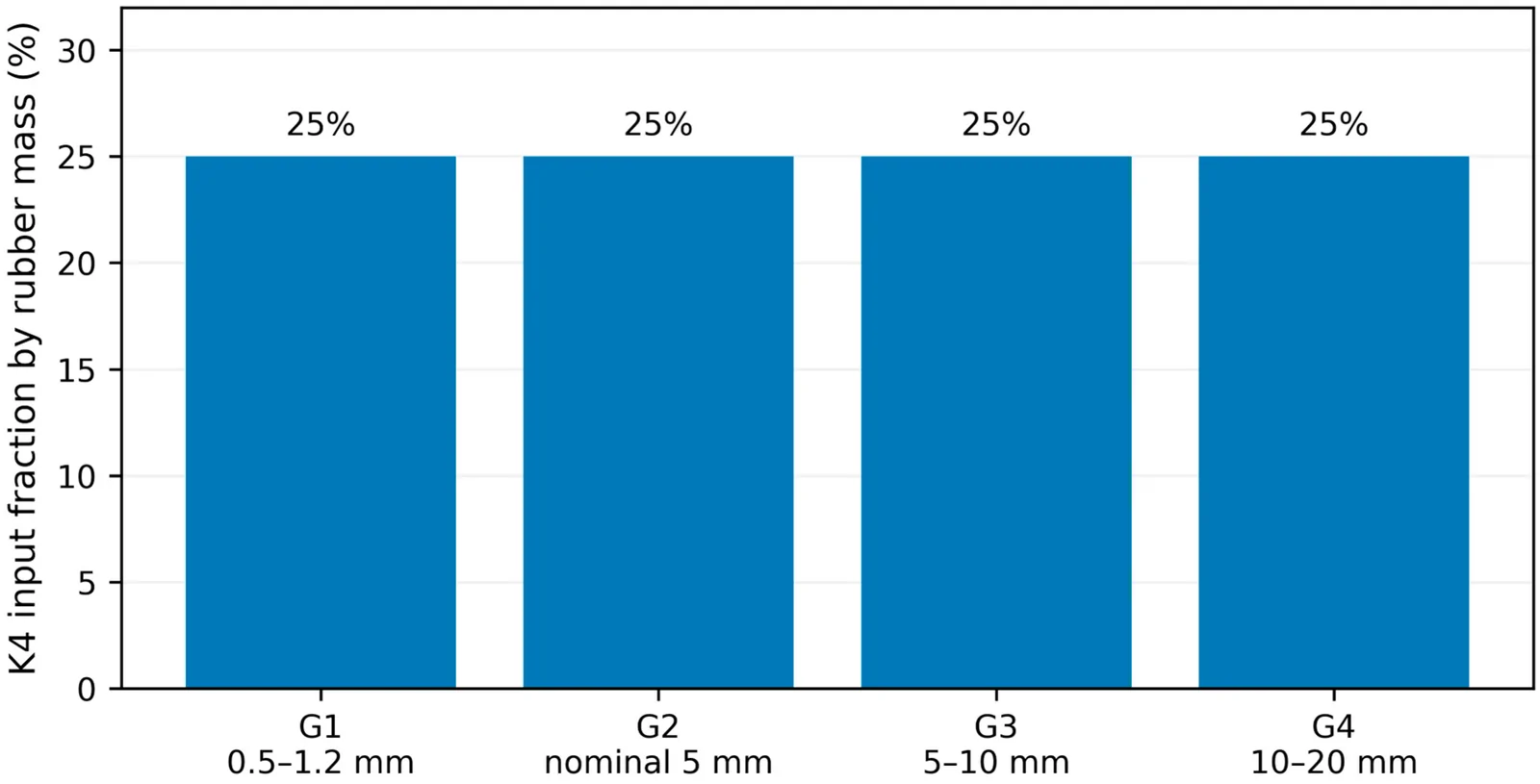
Nominal mass shares of the four rubber fractions in K4 (a formulation schematic, not a measured sieve-analysis curve).

The experimental program comprised four matrix systems (polyurethane, epoxy, novolac–hexamine, and unsaturated polyester) and four rubber gradations (K1–K4), corresponding to 16 distinct formulation combinations—not 16 independent replicates of one material (Figure 1). The G1 fraction was 0.5–1.2 mm, G2 was nominally 5 mm (no verified sieve limits), G3 was 5–10 mm, and G4 was 10–20 mm. K1 contained G1 only; K2, G2 only; K3, G1 and G2 at 50:50 by mass; and K4, G1–G4 at 25% each. The K4 distribution illustrated in Figure 2 represents nominal input fractions, not a measured sieve-analysis passing curve.

### 2.2. Polymer Matrix Formulations

For the compression/UPV series, each recipe contained 800 g of rubber. Polyurethane (CREANATE 110-10 A) (Flokser Chemical Industry and Trade Inc., Istanbul, Turkey) contained 160 g of a one-component MDI-based binder. Epoxy (CREACASE EPNT 350) (Flokser Chemical Industry and Trade Inc., Istanbul, Turkey) contained 160 g of component A and 80 g of component B (100:50 by resin mass). Novolac (GENFEN 9025P) (Gentas Chemical Industry Trade and Marketing Inc., Kocaeli, Turkey) contained 160 g of resin and 16 g of additional hexamine; the commercial novolac also contained hexamine according to its technical data. Polyester (TP100) contained 160 g of resin, 6.4 g of MEKP, and 0.64 g of cobalt accelerator. Therefore, the nominal 20% ratio denotes main resin relative to rubber—not total reactive mixture. Including auxiliary components, the respective binder-system/rubber ratios were 20.00%, 30.00%, 22.00%, and 20.88%. For flexural specimens the corresponding recipe basis was 600 g rubber, with component masses scaled proportionally (Table 1).

### 2.3. Mixing, Molding, Compaction, and Curing

Rubber fractions and matrix components were weighed separately according to the formulation. The relevant reactive resin components were prepared according to their recipes (Table 2) and combined with rubber; mechanical mixing was applied for a fixed 3 min and was terminated when visually homogeneous binder coverage was observed. Recorded mixer speed and an independently verified mixing temperature are not available and therefore are not reported as controlled values. Metal and MDF mold surfaces were cleaned and treated with a wax-based release agent. Mixtures were placed progressively, covered with matching plates and compressed/fixed using clamps.

In preliminary molding, a hydraulic-press gauge reading of approximately 15 bar (1.5 MPa hydraulic-system indication) produced a height reduction of approximately 20 mm in a reference mold. Subsequent clamp-based compaction aimed to approach and maintain that geometrical reduction; the pressure imposed directly on specimens by the clamps was not measured and must not be equated to the gauge reading. Polyurethane, epoxy, and polyester specimens developed demoldable initial physical integrity within approximately 24 h; elevated-temperature post-curing was not applied to these systems. Novolac–hexamine specimens were held in the clamped molds in an oven at 160 °C for 24 h. The specimens were not hot pressed at 2 MPa during this oven treatment. The 28-day age was adopted as a common mechanical comparison time, not as evidence that all matrices require a 28-day cure.

### 2.4. Mechanical Tests and Derived Quantities

Compression and UPV specimens were prepared in 70 × 70 × 70 mm metal molds, with measured final dimensions of approximately 70 × 70 × 50 mm after compaction. In the recorded compression setup, loading acted on the 70 × 50 mm face, giving a nominal initial area of 3500 mm^2^ and a loading-direction initial length of 70 mm. Thus, nominal stress was calculated as σ = F/3500 mm^2^ and apparent strain as εapp = Δu/70 mm, with Δu the testing-machine displacement. The machine files initially used a 50 mm height for strain processing; the 13 recorded curves were reprocessed using 70 mm. Because no specimen extensometer was employed, εapp, Esec,p = σpeak/εapp,peak, and the area Ut = ∫σdεapp to the final recorded point are apparent, device-specific indicators, not a Young modulus, fracture toughness, or measured dissipated energy.

For three-point bending, 160 × 40 × 40 mm prismatic specimens were prepared using the same four-by-four formulation design. Compression and bending methods were informed by ISO 604 [28] and ASTM D695 [29], and ISO 178, respectively, as methodological references; conformity with all requirements of those standards is not asserted for these nonstandard specimen geometries. The test-specific compression loading rate, bending support span, and bending loading rate are not documented sufficiently for numerical reporting. Consequently, the reported nominal strength values should be interpreted as measurements under the recorded laboratory configurations, rather than certified standardized product properties.

### 2.5. UPV and Selected Characterization

Direct-transmission UPV measurements used coupling gel and the measured pulse path length (V = L/t), with EN 12504-4 and ASTM C597 [30] as measurement-principle references rather than claims of full standard compliance for polymer–rubber materials. For SEM analysis, the authors selected specimens preferentially from the mechanically strongest compression formulations within each binder series. The 28-day data identify these selection targets as epoxy–K1 (8.21 MPa), polyurethane–K4 (6.29 MPa), novolac–hexamine–K2 (6.29 MPa), and polyester–K4 (1.35 MPa). This purposeful selection, confirmed by the authors, is distinct from randomly sampling all 16 formulations. SEM images were acquired with a JEOL JSM-6510 (20 kV, secondary electron mode) (JEOL, Tokyo, Japan). Not every original individual image file retains an independently checkable K-code label, so the specified K groups refer to the reported sampling strategy and compression rankings, rather than independent traceability of every micrograph. EDS spectra and element maps characterize selected local regions, with 60 s and 1443.8 s live times reported for spectral and mapping records, respectively; a one-to-one correspondence between every EDS field and a K-labeled SEM field is not asserted. ATR-FTIR spectra (PerkinElmer Spectrum 100, 4000–400 cm^−1^, 4 cm^−1^ resolution) (PerkinELMER, Waltham, MA, USA) predominantly characterize starting rubber and matrix materials, not quantitative interfacial chemical bonding.

For representative TGA/DTG, PerkinElmer STA 6000 (PerkinELMER, Waltham, MA, USA) data for epoxy, novolac–hexamine and polyurethane document nitrogen flow of 20 mL/min, 30–800 °C and 20 °C/min heating. The polyester temperature/mass profile was digitized approximately from an instrument graph because its original numeric record was not available; its complete heating program was not independently verified. TGA/DTG was not performed on all 16 mechanical combinations. The analyzed epoxy and novolac files identify fine-rubber representative material, while the polyurethane and polyester K codes cannot be fully confirmed. Results should not be generalized to untested gradations or used to infer fire resistance.

### 2.6. Test-Age Selection and Statistical Scope

Trial formulations used 10%, 15%, and 20% main-resin/rubber ratios. The 10% assessment was primarily a molding/initial-cohesion observation, and quantitative mechanical comparisons in the available records center on selected 15% and 20% recipes. A working target of approximately 5–10 MPa compression was defined for prospective non-load-bearing applications, not as a universal regulatory requirement. Some 15% formulations already met the lower end of this target. The 20% formulation was selected as a common level for the subsequent comparative program because several candidates reached the project target and avoiding additional resin use was an intended design consideration. Neither cost minimization nor environmental superiority was quantified; no recipe above 20% was tested, so 20% is not an established optimum. UPV–strength relationships use four gradations per binder (n = 4 data pairs, not experimental replicates) and are descriptive/exploratory only.

### 2.7. Preliminary Binder Content Screening and Reference-Recipe Selection

In preliminary formulation screening, main-resin contents of 10%, 15%, and 20% of rubber mass were considered. The 10% batches were evaluated during preliminary production and are not supported by the same quantified mechanical dataset as the 15% and 20% comparisons. A working 5–10 MPa compressive-strength range was a project-specific target for prospective non-load-bearing uses, not an across-the-board Turkish product-standard limit. Several epoxy and novolac combinations reached at least 5 MPa with 15% main resin, while selected 20% formulations reached 5–8.21 MPa. We retained 20% main resin relative to rubber as a common reference for subsequent cross-formulation comparisons in keeping with the intent to limit additional resin consumption; however, no formulations above 20% were tested, total reactive contents were not identical across matrices, and neither the lowest sufficient dosage for every formulation nor any quantified economic/environmental optimum was established.

## 3. Results and Discussion

The 28-day reference compression/flexure matrix revealed formulation-specific rankings. Epoxy–K1 reached 8.21/2.21 MPa, polyurethane–K4 reached 6.29/1.94 MPa, and novolac–K2 reached 6.29/1.38 MPa. Polyester values were lower in the tested formulation (1.22–1.35 MPa compression; 0.20–0.24 MPa flexure). These comparisons reflect each complete recipe and processing procedure, including unequal total reactive-component ratios and different curing conditions. They therefore do not isolate matrix chemistry as the only causal variable. Compression-derived apparent deformation parameters were recalculated for a 70 mm loading length: epoxy–K1 had Ut = 1.968 MJ/m^3^, polyurethane–K4 Ut = 1.581 MJ/m^3^ with εapp,peak ≈ 0.491, and novolac–K3 the largest Esec,p = 55.086 MPa but an Ut of 0.326 MJ/m^3^ over its shorter recorded deformation range. UPV patterns differed by system; trends based on four gradations per binder are exploratory, not prediction equations. Selected microscopic and thermal measurements are discussed at the level of their verified sample identities and method-specific evidence.

### 3.1. Mechanical Performance: Matrix Formulation and Gradation

**Figure 3:**
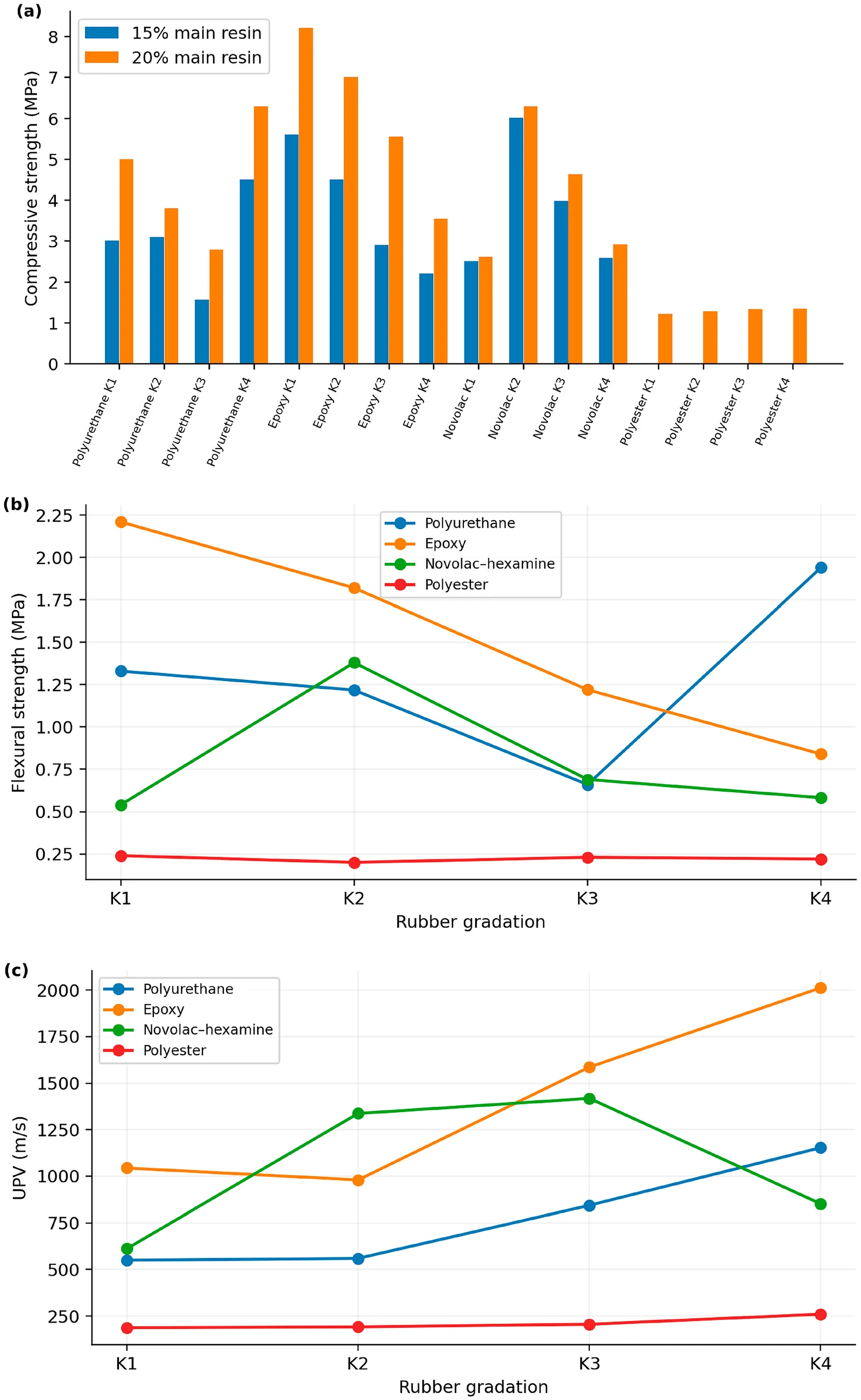
Enlarged nonredundant plots: (**a**) preliminary 15% versus 20% compression; (**b**) 28-day flexural strength; (**c**) 28-day UPV across K1–K4.

The available compression and flexure data vary with both matrix formulation and rubber gradation (Table 3 and Figure 3). Within the tested recipes, epoxy–K1 showed the largest mechanical maxima, polyurethane–K4 was the strongest polyurethane arrangement, and novolac–K2 was the strongest novolac arrangement. These are within-program rankings, not universally optimized recipes. A finer fraction can increase matrix–rubber contact area while raising coverage demand, whereas a broad distribution may assist packing but also introduce voids or heterogeneous contacts. The study does not independently quantify wettability, void fraction, interfacial adhesion, or degree of cure; accordingly, such factors are considered possible explanations rather than directly verified causes. Apparent formulation differences also reflect auxiliary-component loadings and the oven-cured novolac process. Polyester exhibited the lowest mechanical values under the investigated recipe and showed local heterogeneous/open-void morphologies; the presently available SEM/EDS observations do not demonstrate free-radical scavenging or polymerization inhibition. Published polyester–rubber studies associate mechanical changes with rubber loading, gradation and interfacial quality [11,15,16,17], providing a relevant but not identical comparison.

The auxiliary 15–20% comparison suggests increasing compression strength for several tested recipe–gradation combinations, but trial ages and replicate information are not uniformly available for a controlled dose–response analysis. Since some 15% formulations reached at least 5 MPa and no contents above 20% were evaluated, 20% is presented as a selected reference ratio, not a measured economic, environmental, or mechanical optimum. Data in Table 4 should be read with this formulation scope in mind.

### 3.2. Ultrasonic Pulse Velocity and Exploratory Strength Relationships

The UPV values differed across matrix systems and rubber gradations (Table 3), but UPV alone did not rank compressive strength consistently. For example, epoxy–K4 showed 2012 m/s UPV but 3.54 MPa compression, whereas epoxy–K1 showed 1044 m/s and 8.21 MPa. Wave transmission depends on stiffness, density, heterogeneity, interfaces, contacts, and measurement configuration; the present results do not establish a unique cause for each trend.

$$\mathrm{UPV}=({\frac{M}{\rho })}^{1/2}$$

For each binder, Pearson coefficients were calculated using only four *different formulation/gradation points* (n = 4), not four independent replicates of one treatment. Such small groups can yield unstable coefficients and cannot justify predictive calibration or statistical generalization. The analysis is retained as exploratory trend visualization. No externally validated UPV-based strength estimator is proposed.

**Figure 4:**
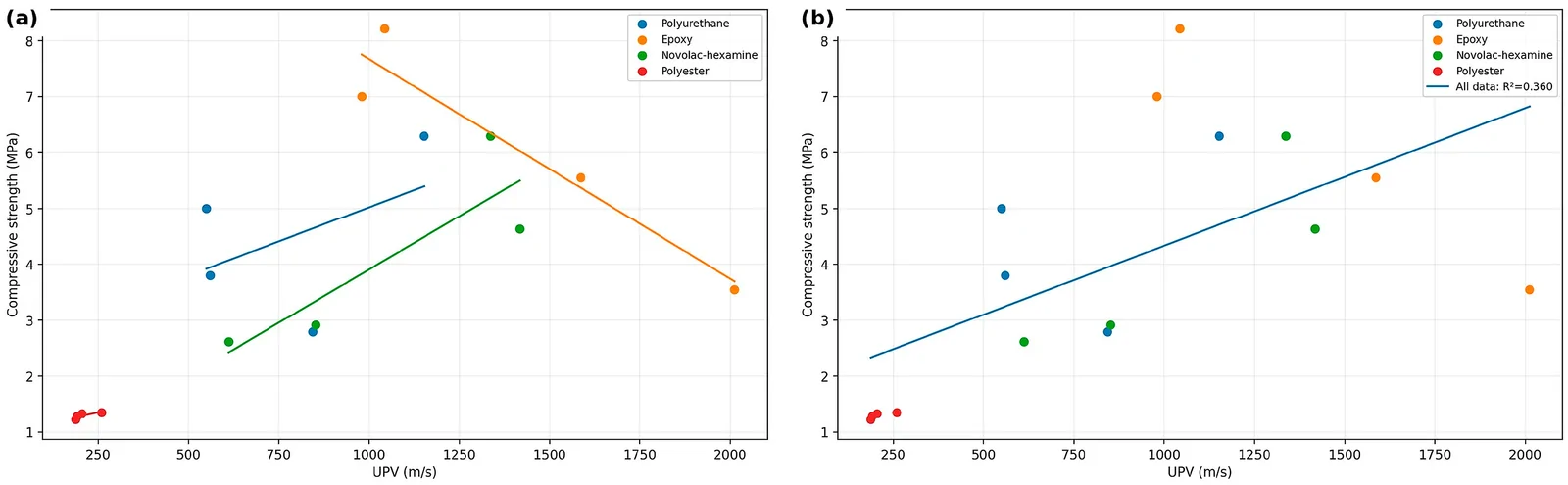
UPV–compressive strength relationships: (**a**) binder-specific trends; (**b**) all data combined.

Across all 16 formulations, the apparent UPV–compressive strength correlation was r = 0.600 (R^2^ = 0.360). After subtracting each binder-group mean from the corresponding variables, the pooled association was r = −0.078 (R^2^ = 0.006). The difference highlights formulation-group effects in this small dataset rather than demonstrating a broadly applicable material law (Figure 4).

### 3.3. Apparent Compression Stress–Strain Response and Loading Work

**Figure 5:**
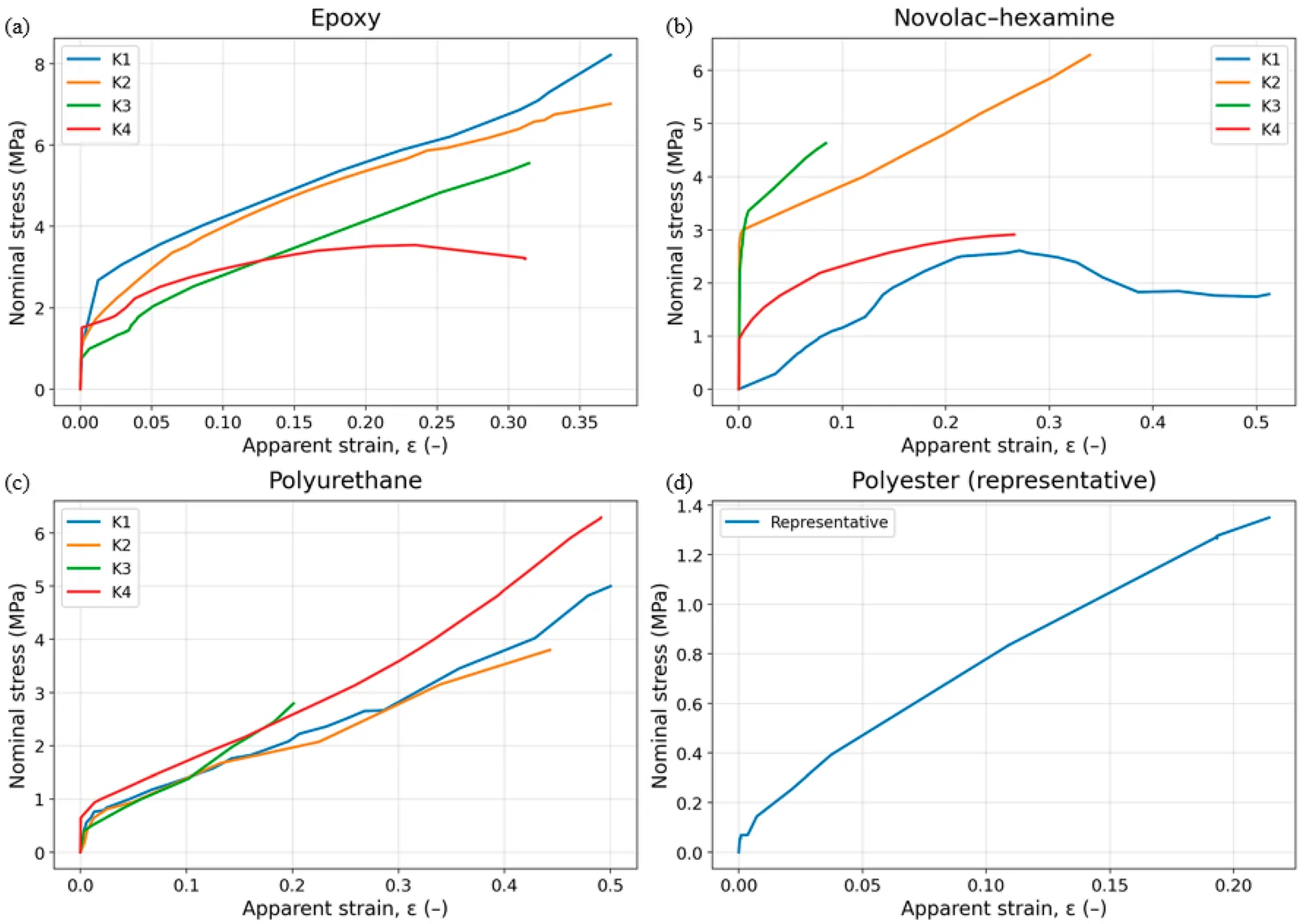
Apparent compressive stress–strain curves recomputed for a 70 mm loading-direction length from machine displacement: (**a**) epoxy, (**b**) novolac–hexamine, (**c**) polyurethane, and (**d**) representative polyester. For readability, the updated plots are presented as continuous line curves without point markers.

**Figure 6:**
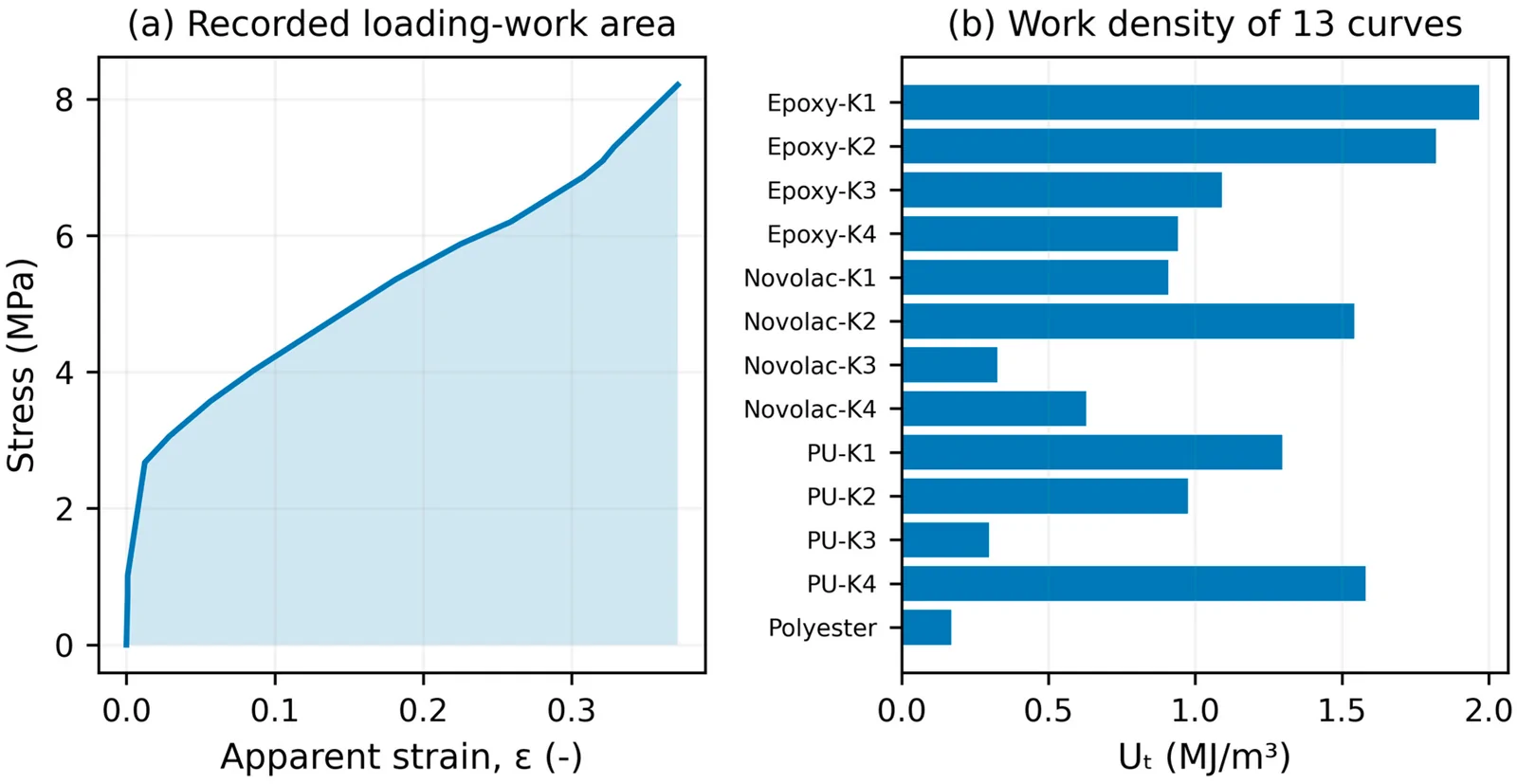
(**a**) Representative apparent compression loading-work area and (**b**) calculated work densities of the 13 available curves (h0 = 70 mm).

**Figure 7:**
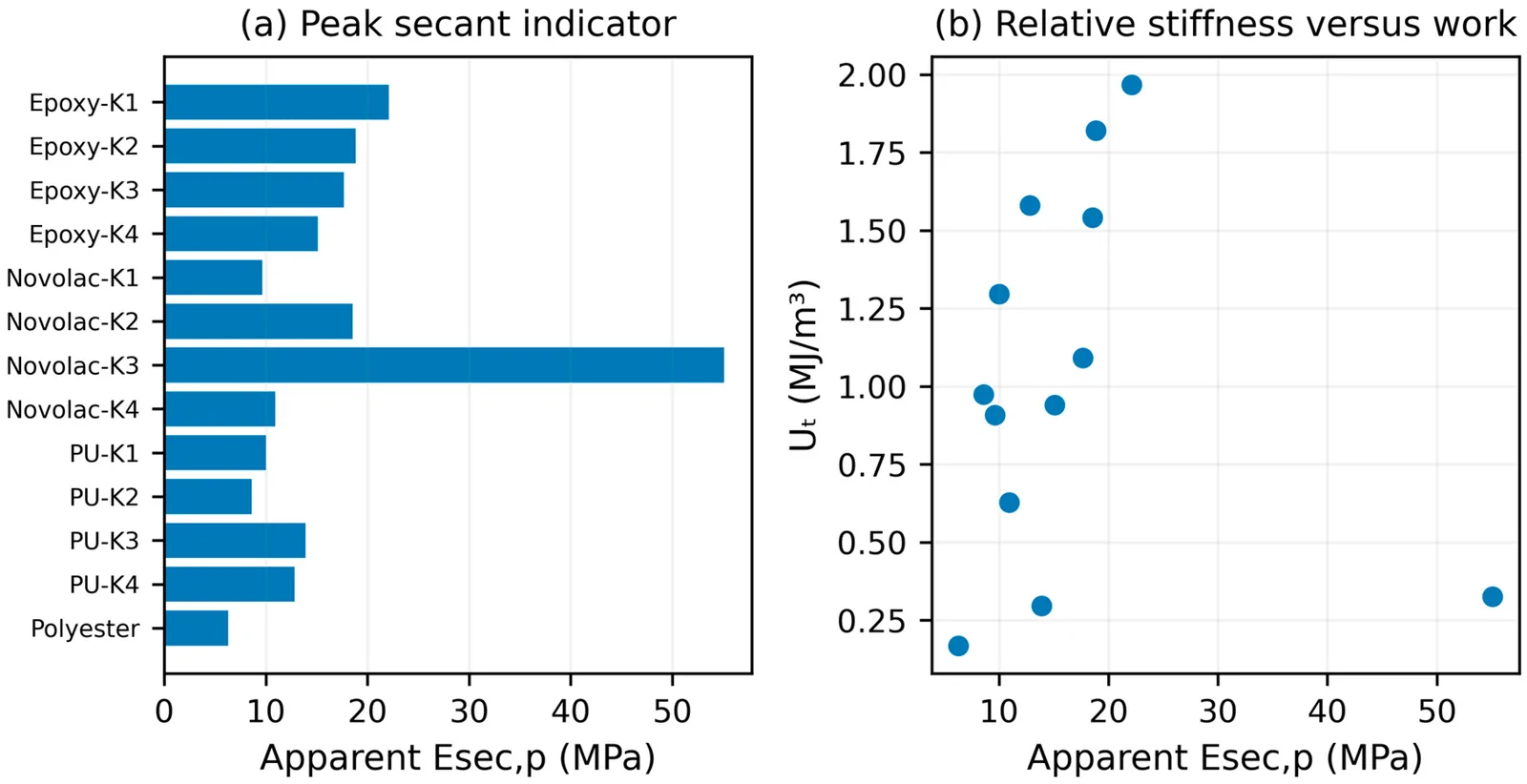
(**a**) Apparent peak secant modulus and (**b**) secant modulus versus integrated apparent work density for the 13 available curves.

The 13 available recorded stress–strain trajectories (four per epoxy, polyurethane, and novolac system; one polyester representative) were reprocessed using the actual 70 × 50 mm loading face and a 70 mm loading-direction initial length. Apparent strain is based on crosshead displacement, which includes possible specimen seating and equipment compliance. Consequently, the resulting peak secant slopes are relative indicators, and curve areas represent work input up to varying recorded end points, not measured damping or fracture toughness. Results are listed in Table 5 and Table 6 and shown in Figure 5, Figure 6 and Figure 7.

The main observed responses were as follows:

Polyurethane–K4 achieved a nominal stress maximum of 6.29 MPa at εapp ≈ 0.491 and Ut ≈ 1.581 MJ/m^3^. Its response extends across a comparatively broad recorded displacement range, but this single loading path does not establish recoverable elasticity, cyclic damping or long-term permanent set.

Epoxy–K1 reached 8.21 MPa at εapp ≈ 0.371, with the largest integrated apparent loading-work density among the available curves, Ut ≈ 1.968 MJ/m^3^. For epoxy–K2, the reported reference compressive stress is 7.000 MPa, and the corresponding apparent reference peak secant indicator is Esec,p ≈ 18.846 MPa at εapp ≈ 0.371. Its work integral, calculated from the original recorded curve trajectory without changing the measured displacement points, is approximately 1.821 MJ/m^3^.

Novolac–K3 reached the largest apparent peak secant modulus, Esec,p ≈ 55.086 MPa, but its recorded curve ended at εapp ≈ 0.084 and Ut ≈ 0.326 MJ/m^3^. The short recorded range and machine-derived displacement prevent classifying this response conclusively as intrinsic brittle fracture or an extensometer-based Young modulus. Novolac–K2 yielded the largest compression result within that binder system.

The representative polyester curve reached 1.35 MPa at εapp ≈ 0.214, with Ut ≈ 0.169 MJ/m^3^. Its relatively low work density is consistent with the lower compression/flexure results under this formulation, but it does not by itself identify a particular chemical curing deficiency. The polyester curve was not reliably linked to one of the four particle-size codes.

#### Prospective Application Context

The recorded compression strengths, apparent displacements, and curve integrals can guide subsequent product-oriented testing, but the present measurements do not verify dynamic damping, seismic isolation, impact performance, fatigue endurance or fire resistance. Potential uses must therefore remain hypothetical until assessed against an identified product standard and service conditions.

- Polyurethane-rich formulations may merit further study as flexible tile or impact-attenuation materials. Tests of cyclic compression, permanent deformation, resilience, abrasion, slip resistance and aging would be required before a specific product claim.
- Epoxy–K1 showed comparatively high measured static compressive/flexural strength and recorded work density. Modular protective components are possible research directions, subject to impact, fatigue, weathering and safety testing; crashworthiness or semi-structural suitability was not established.
- Novolac formulations showed gradation-dependent apparent stiffness and comparatively high residue in representative nitrogen TGA measurements. Rigid non-load-bearing panel research would require dimensional stability, controlled-cure assessment and standardized fire reaction and mechanical testing. High TGA residue is not a fire-resistance classification.
- For the tested polyester formulation, further work could assess surface compatibility, void control, matrix composition and cure degree. These are hypotheses for subsequent studies rather than conclusions that chemical radical scavenging has been demonstrated.

### 3.4. Selected SEM/EDS Morphology and Elemental Distribution

**Figure 8:**
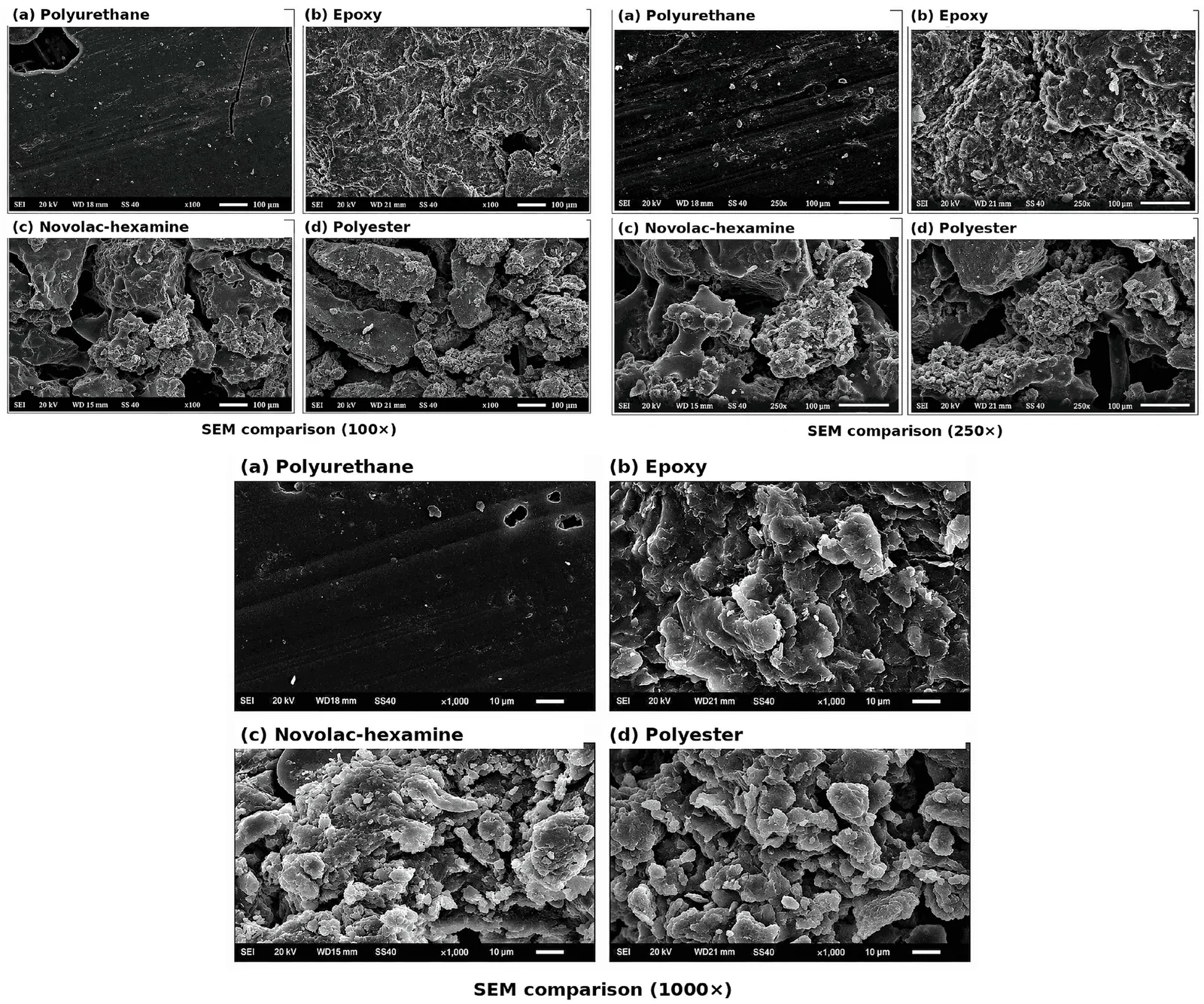
Selected low- to medium-magnification SEM images. Author-reported selection targets were the highest-compression series for each matrix: epoxy–K1, polyurethane–K4, novolac–K2 and polyester–K4. Individual micrograph-to-K-code device provenance was not complete for all exports.

**Figure 9:**
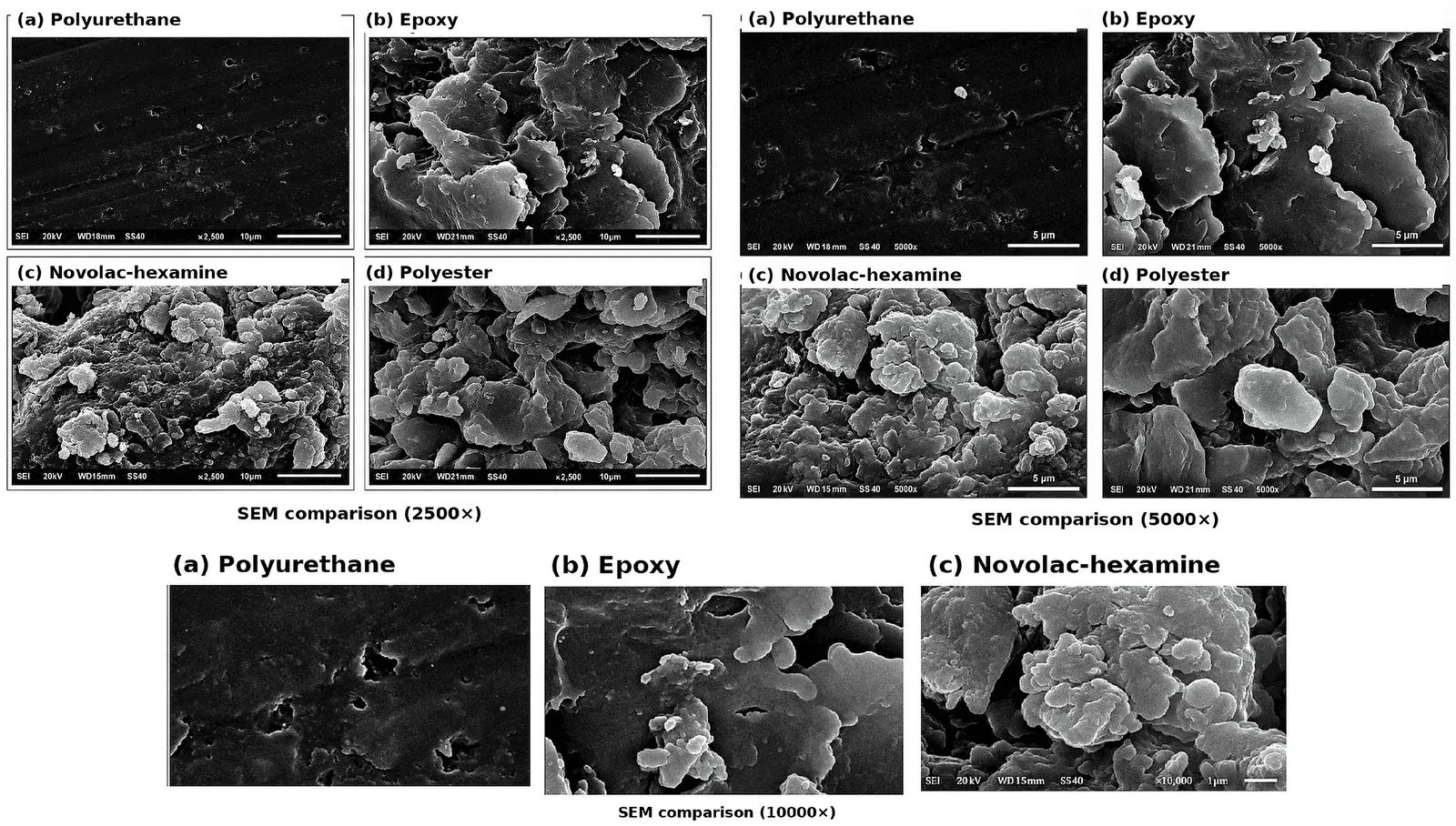
Selected higher-magnification SEM fields from the author-reported high-compression specimen selections (epoxy–K1, polyurethane–K4, novolac–K2 and polyester–K4). The microscopic fields are local observations, not full-formulation statistically representative images.

The authors deliberately selected SEM specimens from the highest reported 28-day compressive-strength combinations for the respective matrix systems (epoxy–K1, polyurethane–K4, novolac–hexamine–K2, and polyester–K4; Table 3). This selection describes specimen-screening intent, not full-factorial image sampling or proof that each exported SEM field can be linked individually to a surviving K-labeled device record. Figure 8 and Figure 9 therefore illustrate the selected high-strength specimen groups as reported by the authors, subject to that field-level traceability limitation. SEM reveals local surface/fracture morphology; EDS describes semi-quantitative elemental signals in selected analysis locations. Neither method alone measures adhesive strength, porosity, conversion, or a specific covalent-bond formation mechanism.

The epoxy set was selected with epoxy–K1 as the highest-compression formulation (8.21 MPa). Its displayed fracture micrographs contain rough, complex regions that may be consistent with heterogeneous crack paths. Because image-level instrument identifiers are not uniformly complete, these observations are qualitative and do not establish a direct quantitative K1 structure–strength relationship or a measured interfacial adhesion value.

The polyurethane imaging selection was guided by the strongest polyurethane compression group, K4 (6.29 MPa). The selected fields include relatively continuous matrix regions with local discontinuities. The observations are compatible with heterogeneous deformable materials, but their local appearance cannot itself explain the K4 mechanical maximum or establish cyclic damping behavior.

The novolac imaging selection targeted K2, which had the highest recorded compression strength within the novolac–hexamine group (6.29 MPa). Micrographs show granular/clustered surface features, but image texture alone does not quantify stiffness or degree of cure. Notably, the highest apparent peak secant indicator came from a different combination, novolac–K3; it must not be attributed to the imaged K2 selection.

For polyester, the highest-compression formulation within the studied series was K4 (1.35 MPa), and the authors describe this as the SEM selection target; polyester–K1 instead gave the largest flexural result (0.24 MPa). The selected micrographs display local heterogeneous/open-void regions that may be associated with the comparatively low mechanical performance. Direct polyester–scrap tire experiments report strength loss and interface effects [15,16,17], and work on styrene polymerization in the presence of ground tire rubber reports altered radical-reaction kinetics [31,32]. Importantly, those latter studies concern styrene polymerization systems, not direct cure measurements on the present TP100–MEKP–cobalt specimens. Thus, cure inhibition remains a plausible but untested explanation; SEM/EDS does not demonstrate it.

**Figure 10:**
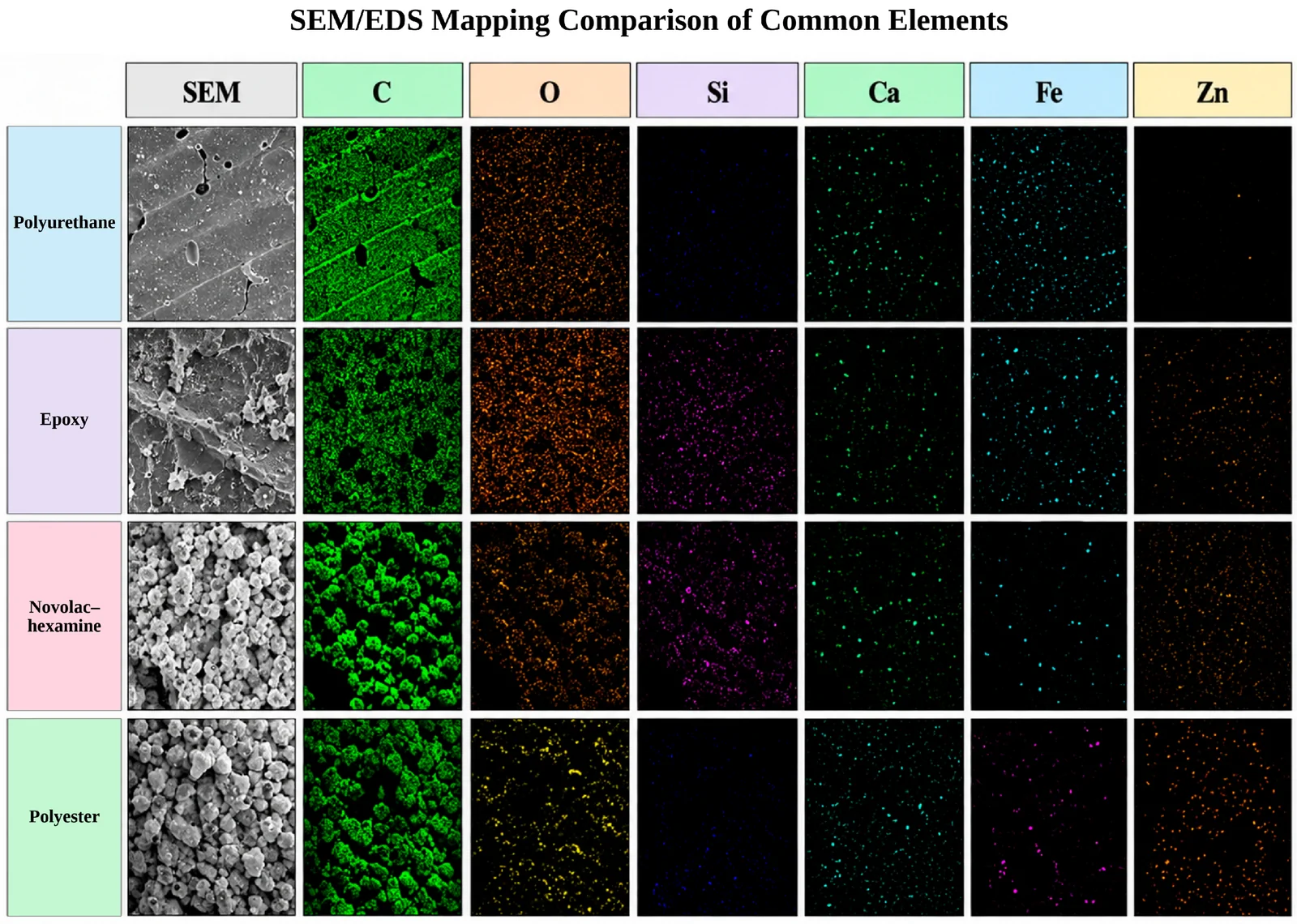
Selected local SEM fields and EDS elemental maps of polyurethane, epoxy, novolac–hexamine, and polyester. The panels show the distributions of C, O, Si, Ca, Fe, and Zn available in the original instrument outputs. Exact matching of every analysis position to an individual K-labeled specimen field could not be independently confirmed.

The elemental maps (Figure 10) and semi-quantitative EDS results (Table 7) represent selected regions of the polymer–rubber materials, not paired measurements at standardized identical positions across all formulations. Zn, Si, O and other detected elements can reflect rubber compounding and filler chemistry; local weight percentages depend on the sampling region and should not be interpreted as direct interfacial coverage percentages or measures of chemical reaction.

The comparatively different local Zn and Si signals across selected EDS fields are descriptive. They cannot prove that a given additive remained chemically exposed, scavenged radicals, reduced polyester crosslinking, or caused measured strength changes. Matched specimen/region sampling, controlled chemical cure analysis (e.g., calorimetry or spectroscopy of reacted composite interfaces) and mechanical interface tests would be needed to evaluate these hypotheses.

### 3.5. ATR-FTIR Fingerprints of Selected Constituents

**Figure 11:**
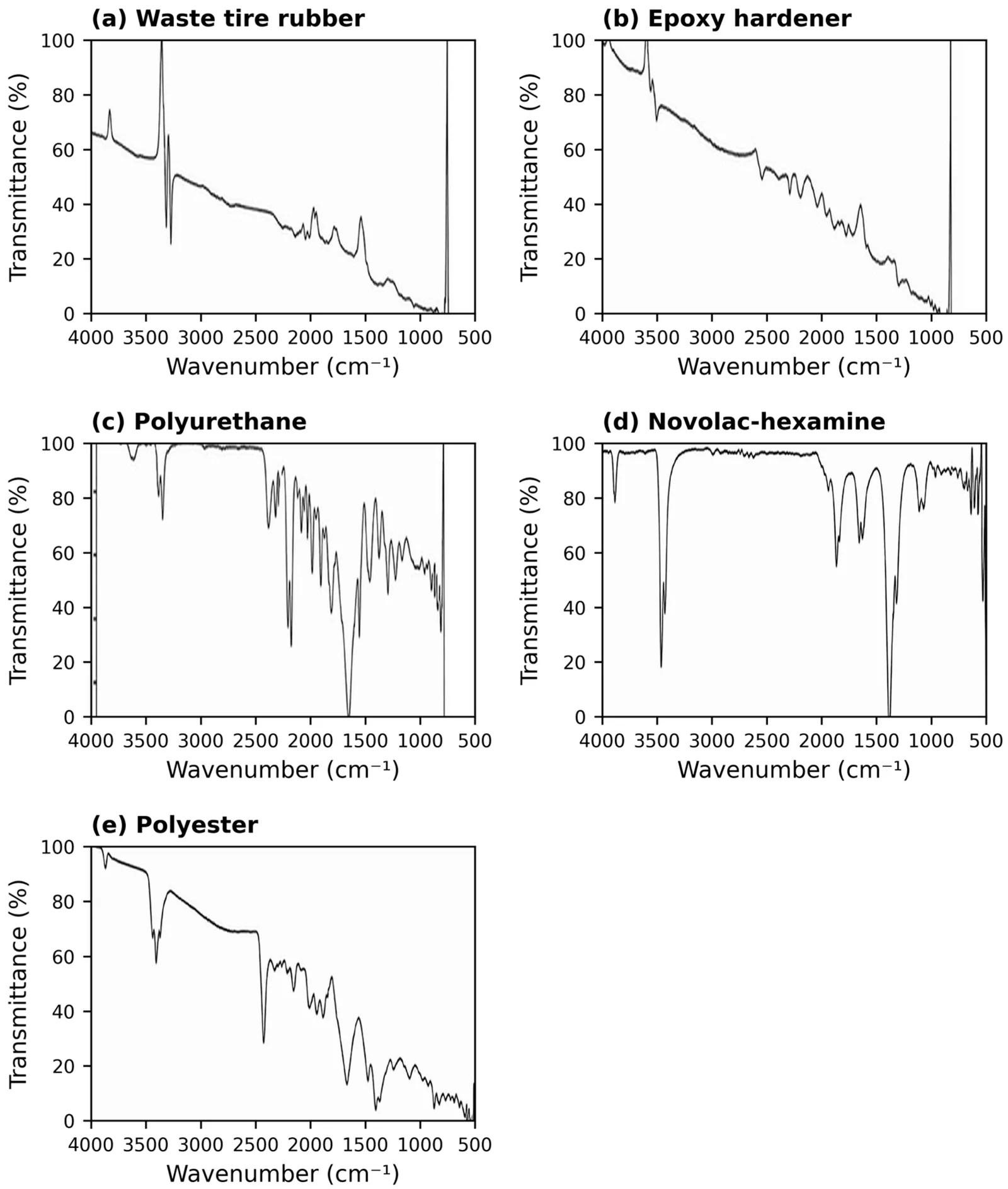
ATR-FTIR spectra of selected rubber and matrix constituents; spectra were not obtained for matched interfaces across all 16 formulations.

Attenuated total reflectance Fourier-transform infrared spectroscopy (ATR-FTIR) was used to compare the chemical fingerprints of selected constituent samples, including raw rubber, an epoxy hardener, polyurethane, novolac–hexamine and polyester (Figure 11). Instrument records cover 4000–400 cm^−1^ at 4 cm^−1^ resolution. The available dataset is not a matched before/after cure study on all 16 composite interfaces; therefore no specific rubber–matrix covalent reaction, grafting efficiency, or cure inhibition can be inferred from these spectra alone.

- The polyurethane spectrum includes bands consistent with the chemistry of the supplied one-component prepolymer. Because the tested FTIR specimen was not a fully paired cured composite, band positions are interpreted as fingerprints rather than a quantitative conversion measure.
- Novolac–hexamine exhibits spectral features compatible with its phenolic/aromatic functional groups. A detailed degree-of-cure assignment would require appropriate reference samples or an independent reaction analysis.
- The polyester raw material exhibits characteristic ester-associated absorption bands. Its spectrum confirms the type of matrix constituent, not whether polymerization within a rubber-rich composite was inhibited.

Taken together, the constituent spectra are useful for documenting relevant starting chemistry but do not prove or exclude new covalent bonds at individual rubber–polymer interfaces. The mechanical trends are therefore discussed primarily as empirical formulation-specific observations.

### 3.6. Representative TGA/DTG Thermal Degradation Behavior

**Figure 12:**
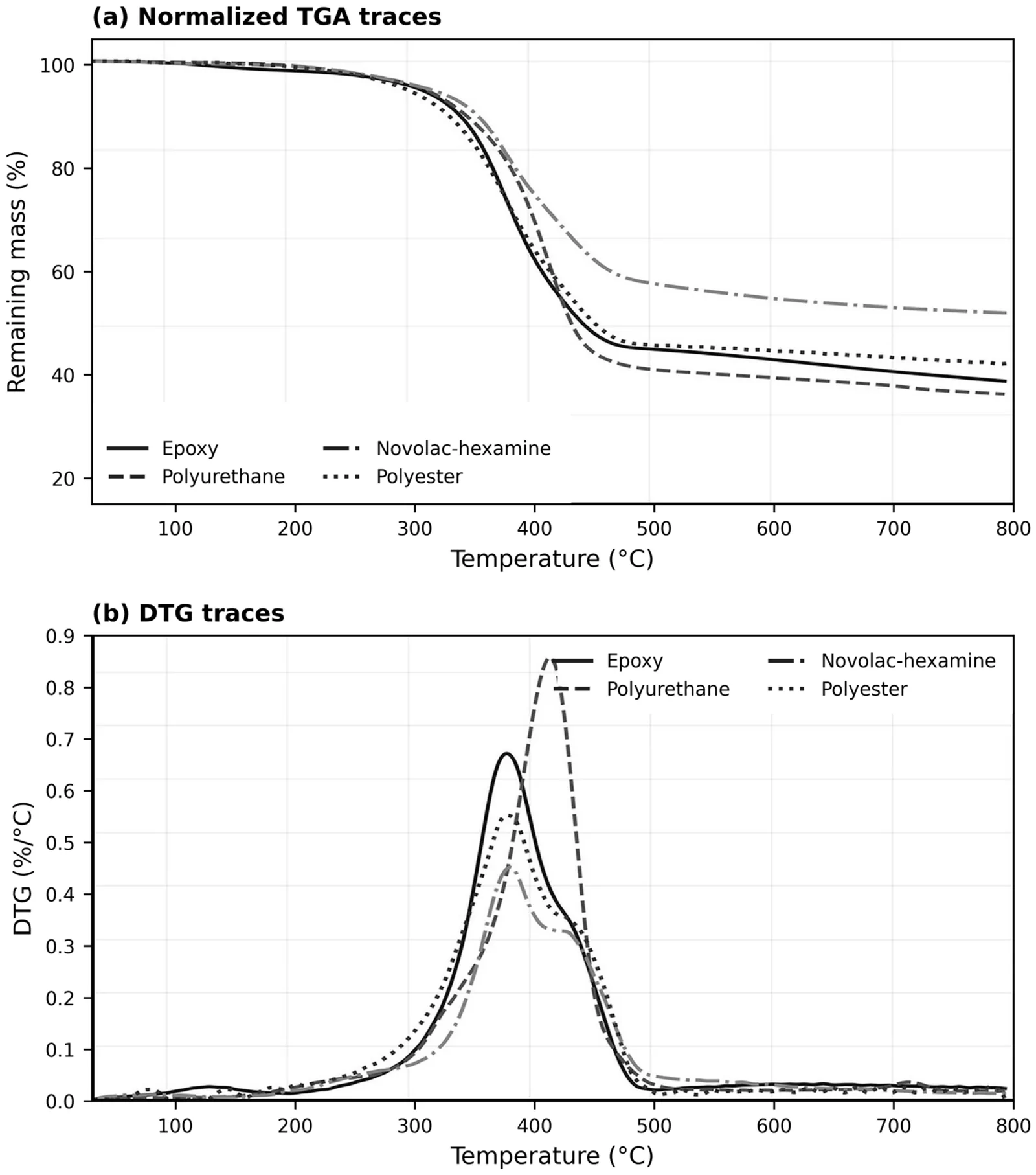
Representative (**a**) normalized TGA and (**b**) DTG traces. Polyester profile is graph-digitized and approximate.

The selected thermal records (Figure 12; Table 8) describe mass-loss profiles during controlled heating, mainly under nitrogen. They do not represent testing of all 16 mechanical formulations, and the polyester profile was reconstructed approximately from a recorded instrument graph. Among the available numerical profiles, novolac–hexamine showed the highest listed 10% mass-loss temperature and the largest final remaining mass (approximately 43.1%); polyurethane showed the highest DTG peak temperature (approximately 417.0 °C). Differences in measured residues may reflect multiple rubber, matrix, carbonaceous, and inorganic constituents rather than a single quantified shielding mechanism.

The polyester representative record showed comparatively low early mass-loss temperatures, but its digitized nature and incomplete program metadata limit precision. The combined microscopy and TGA observations cannot demonstrate reduced polyester crosslink density or identify radical scavenging as the cause of thermal and mechanical differences.

Thermal mass-loss rankings under nitrogen are not interchangeable with measured fire safety, flame spread, smoke emission or retention of load-bearing strength at temperature. Those properties require independent and application-specific test methods.

#### Cross-Property Discussion and Limits of Interpretation

The mechanical, ultrasonic, microscopic and thermogravimetric results measure different properties of the investigated formulations. The SEM specimen-selection strategy prioritized each matrix family’s highest 28-day compression group, but the available image-level identifiers are insufficient to infer matched specimen-level causal relationships from every local micrograph. Epoxy–K1 was strongest in the 28-day compression/flexure matrix and had the largest recorded apparent loading-work density; this does not imply superior high-temperature load capacity. The representative novolac TGA profile had relatively high residue and 10% mass-loss temperature, whereas polyurethane had a higher DTG maximum; none establishes certified fire performance. Polyester imaging showed local heterogeneity, but neither SEM/EDS nor constituent ATR-FTIR established chemical curing inhibition. Thus, performance trade-offs are reported with their formulation and method boundaries.

## 4. Potential Applications and Methodological Limitations

Table 9 distinguishes measured material-level observations from potential future applications. The experiments provide a starting basis for studies of flexible surface products, protective components, and rigid non-load-bearing panels; they do not constitute evidence of seismic isolation, railway damping, crashworthiness, fire resistance, or certified construction-product compliance. Cyclic/dynamic loading, impact, aging, fatigue, abrasion, slip resistance, leaching/VOC, and standardized fire-reaction tests should be selected according to the intended product.

The principal limitations include unequal total reactive-component loadings and different curing protocols across matrix systems; unavailable numerical mixer speed, test loading rates and flexural support-span values; 13 crosshead-displacement curves rather than 16 independent curves; only four formulation/gradation pairs per binder for exploratory UPV correlations; author-reported selection of higher-compression specimens for SEM without complete surviving image-level K-code traceability; EDS of unpaired local regions; ATR-FTIR of selected constituents rather than matched reacted interfaces; and TGA/DTG on selected profiles instead of all 16 combinations. A standardized specimen-ID, repeat and measurement protocol will be necessary before mechanistic or statistical generalization.

Future studies should investigate rubber surface treatments, controlled matrix stoichiometry, quantified porosity, and cure kinetics using appropriate controls. Polyester-specific studies may test interface wetting and chemical cure as competing hypotheses instead of assuming that the observed low strength was caused by radical scavenging.

## 5. Conclusions

Four matrix formulations and four rubber gradations were compared using a common nominal main-resin/rubber ratio of 20% and 28-day reference mechanical age. Auxiliary-component masses and thermal treatment differed across systems, limiting isolation of polymer chemistry as the sole cause of performance differences.

- Epoxy–K1 reached 8.21 MPa compression and 2.21 MPa flexure; polyurethane–K4, 6.29 and 1.94 MPa; and novolac–K2, 6.29 and 1.38 MPa. Polyester remained lower under the formulation studied (1.22–1.35 MPa compression). These observations do not establish universal optimal matrices or gradations.
- Using the verified 70 × 50 mm loaded area and 70 mm loading direction, the 13 available machine-displacement-based curves yielded a maximum apparent work density of 1.968 MJ/m^3^ for epoxy–K1 and a maximum peak secant indicator of 55.086 MPa for novolac–K3. The different curve end points and absence of an extensometer limit the material-property interpretation of these figures.
- Four-point-per-binder UPV–strength relationships were exploratory. Authors selected SEM specimens preferentially from the highest-compression combination of each binder series; these images and local EDS data supplied qualitative context, while constituent ATR-FTIR and representative TGA/DTG documented separate chemistry and mass-loss behavior. Incomplete device-level K-code provenance and the selective nature of characterization limit direct formulation-level causal claims. None of these tests established a polyester cure-inhibition mechanism or certified the composites for fire, seismic or dynamic applications.
- The reported formulation-specific differences provide a basis for better-controlled future studies of matrix composition, rubber gradation, interface treatments, cure degree, durability and application-specific mechanical and fire performance.

## Figures and Tables

**Table 1:** Raw materials and binder systems used in the study.

Material	Product/Key Properties	Supplier	Function
Waste tire rubber granules	G1: 0.5–1.2 mm; G2: nominal 5 mm; G3: 5–10 mm; G4: 10–20 mm	AKO ELT Recycling/AKO Tire Retreading	Main granular rubber phase
Polyurethane	CREANATE 110-10 A; one-component MDI-based prepolymer; density 1.10 ± 0.10 g/cm^3^; viscosity 6750 ± 750 mPa·s; NCO 9.5 ± 0.5%	FLOKSER Kimya	Flexible polymer binder
Epoxy	CREACASE EPNT 350 A + B; two-component system; recommended A:B = 100:50 by mass	FLOKSER Group	Rigid polymer binder
Novolac–hexamine	GENFEN 9025P novolac resin; free phenol < 0.3%; melting range 90–95 °C; intrinsic hexamine 8.50–9.50%; additional hexamine used	GENTAS Kimya	Phenolic binder system
Unsaturated polyester	TP100 orthophthalic casting polyester; MEKP and cobalt accelerator used according to recipe	Turkuaz Polyester	Polymer binder

**Table 2:** Rubber gradation codes and main mix recipes (20% nominal main resin by rubber mass; auxiliary components shown separately).

Code/System	Rubber Phase	Mass Ratio/Main Binder	Additional Component(s)
K1	G1 only	100% fine fraction	800 g G1 for the compression/UPV/stress–strain series
K2	G2 only	100% medium fraction	800 g G2
K3	G1 + G2	50% + 50%	400 g G1 + 400 g G2
K4	G1 + G2 + G3 + G4	25% each	200 g of each fraction
Polyurethane	800 g rubber granules	160 g CREANATE 110-10 A	None
Epoxy	800 g rubber granules	160 g EPNT 350 A	80 g EPNT 350 B hardener
Novolac–hexamine	800 g rubber granules	160 g GENFEN 9025P	16 g additional hexamine
Polyester	800 g rubber granules	160 g TP100	6.4 g MEKP + 0.64 g cobalt accelerator

**Table 3:** Compressive strength, flexural strength, and UPV for the 16 main 28-day formulations.

Binder	Particle-Size Arrangement	Compressive Strength (MPa)	Flexural Strength (MPa)	UPV (m/s)
Polyurethane	K1—Fine	5.000	1.330	550
Polyurethane	K2—Medium	3.800	1.218	560
Polyurethane	K3—Fine + medium	2.790	0.660	844
Polyurethane	K4—Four-fraction blend	6.290	1.940	1153
Epoxy	K1—Fine	8.210	2.210	1044
Epoxy	K2—Medium	7.000	1.820	980
Epoxy	K3—Fine + medium	5.550	1.220	1586
Epoxy	K4—Four-fraction blend	3.540	0.840	2012
Novolac–hexamine	K1—Fine	2.610	0.540	612
Novolac–hexamine	K2—Medium	6.290	1.380	1337
Novolac–hexamine	K3—Fine + medium	4.630	0.690	1418
Novolac–hexamine	K4—Four-fraction blend	2.910	0.582	852
Polyester	K1—Fine	1.220	0.240	188
Polyester	K2—Medium	1.280	0.200	192
Polyester	K3—Fine + medium	1.330	0.230	206
Polyester	K4—Four-fraction blend	1.350	0.220	260

**Table 4:** Exploratory UPV–compressive-strength correlations (n = 4 gradations per binder; not independent replicates or validated predictors).

Data Group	n	Pearson r	R^2^	Interpretation
Polyurethane	4	0.461	0.212	Weak-to-moderate positive trend
Epoxy	4	−0.952	0.907	Strong inverse trend within the four gradations
Novolac–hexamine	4	0.866	0.749	Positive trend; mechanical and UPV maxima did not coincide
Polyester	4	0.783	0.613	Positive trend over a very narrow strength range
All data	16	0.600	0.360	Moderate apparent relation affected by polymer clustering
Polymer-centered data	16	−0.078	0.006	No meaningful common linear relation

**Table 5:** Apparent compression parameters from 13 available curves (70 × 50 mm loaded area; 70 mm loading direction).

Series	σmax (MPa)	εpeak (-)	εend (-)	Esec,p (MPa)	Ut (MJ/m^3^)
Epoxy–K1	8.210	0.371	0.371	22.104	1.968
Epoxy–K2	7.000	0.371	0.371	18.846	1.821
Epoxy–K3	5.550	0.314	0.314	17.659	1.091
Epoxy–K4	3.540	0.235	0.312	15.085	0.941
Novolac–K1	2.610	0.271	0.512	9.620	0.908
Novolac–K2	6.292	0.339	0.339	18.551	1.542
Novolac–K3	4.629	0.084	0.084	55.086	0.326
Novolac–K4	2.910	0.266	0.266	10.937	0.628
PU–K1	5.000	0.500	0.500	10.000	1.297
PU–K2	3.800	0.443	0.443	8.581	0.975
PU–K3	2.791	0.201	0.201	13.887	0.297
PU–K4	6.290	0.491	0.491	12.811	1.581
Polyester representative	1.350	0.214	0.214	6.300	0.169

**Table 6:** Integrated summary by matrix system; examples of observed mechanical maxima and selected analyses are not necessarily from the same physical specimens.

Binder System	Best Mechanical Arrangement	Compression/Flexure (MPa)	Stress–Strain/Energy Indicator	Integrated Interpretation
Epoxy	K1—Fine	8.21/2.21	Ut = 1.968; Esec,p = 22.104	Higher static strength and apparent recorded work; local images not K1-paired
Polyurethane	K4—Four-fraction	6.29/1.94	εpeak ≈ 0.491; Ut = 1.581	Large apparent displacement range; damping not directly tested
Novolac–hexamine	K2—Medium	6.29/1.38	Ut = 1.542; Esec,p = 18.551 (K2)	K-dependent compression and stiffness; selected nitrogen TGA
Polyester	K4 for compression/K1 for flexure	1.35/0.24	Ut = 0.169; Esec,p = 6.300 (representative)	Low strength; local heterogeneity (cure mechanism unverified)

**Table 7:** Selected local, semi-quantitative EDS elemental signals (wt.%); not measurements of interfacial reaction or adhesion.

System	O	Si	Ca	Fe	Zn
Polyurethane	23.912	0.724	0.864	1.839	1.495
Epoxy	19.717	3.084	1.202	0.665	3.879
Novolac–hexamine	17.000	2.499	0.574	0.839	7.176
Polyester	17.091	4.107	0.922	1.259	7.400

**Table 8:** Thermal mass-loss and DTG indicators obtained from selected representative samples, not the complete 16-formulation matrix.

Binder System	T5 (°C)	T10 (°C)	DTG Tmax (°C)	Final Residue (%)	Data Status
Epoxy	295.2	334.7	381.5	27.7	Raw numerical data
Polyurethane	299.7	338.3	417.0	24.7	Raw numerical data
Novolac–hexamine	300.3	348.3	384.0	43.1	Raw numerical data
Polyester	≈285.9	≈324.1	≈383.5	≈31.5	Digitized approximately from instrument graph

**Table 9:** Potential product-oriented research directions and required application-specific validation.

System	Dominant Experimental Feature	Potential Direction	Required Validation
Epoxy	High compressive/flexural strength and highest energy area	Low-to-medium-load modular panels, protective layers or rigid covering components	Repeated loading, impact, fatigue, water/UV exposure, fire and scale-up tests
Polyurethane	Wide deformation and high energy absorption	Flexible tiles, sports/playground surfaces, vibration-damping or low-load barrier components	Permanent deformation, abrasion/slip, cyclic loading, VOC/leaching and fire behavior
Novolac–hexamine	Gradation-sensitive stiffness–toughness balance; highest T10 and char residue	Rigid non-structural boards/panels; thermally oriented products after additional validation	Controlled curing, DSC/DMA, LOI/fire tests, thermal cycling and aging
Polyester	Low mechanical level under current interface condition	Reformulation and surface-treatment stage before low-load products	Surface treatment, void control, adhesion, water absorption and repeated mechanical validation

## Data Availability

The tabulated formulation-level results and representative characterization outputs used in this study are provided in the manuscript. Available raw instrument records and additional data can be supplied by the corresponding author upon reasonable request, subject to record availability.
